# Genotyping *Yersinia pestis* in Historical Plague: Evidence for Long-Term Persistence of *Y*. *pestis* in Europe from the 14^th^ to the 17^th^ Century

**DOI:** 10.1371/journal.pone.0145194

**Published:** 2016-01-13

**Authors:** Lisa Seifert, Ingrid Wiechmann, Michaela Harbeck, Astrid Thomas, Gisela Grupe, Michaela Projahn, Holger C. Scholz, Julia M. Riehm

**Affiliations:** 1 Ludwig Maximilian University of Munich, Munich, Germany; 2 State Collection for Anthropology and Palaeoanatomy, Munich, Germany; 3 Bundeswehr Institute of Microbiology, Munich, Germany; University of Texas Medical Branch, UNITED STATES

## Abstract

Ancient DNA (aDNA) recovered from plague victims of the second plague pandemic (14^th^ to 17^th^ century), excavated from two different burial sites in Germany, and spanning a time period of more than 300 years, was characterized using single nucleotide polymorphism (SNP) analysis. Of 30 tested skeletons 8 were positive for *Yersinia pestis*-specific nucleic acid, as determined by qPCR targeting the *pla* gene. In one individual (MP-19-II), the *pla* copy number in DNA extracted from tooth pulp was as high as 700 gene copies/μl, indicating severe generalized infection. All positive individuals were identical in all 16 SNP positions, separating phylogenetic branches within nodes N07_N10 (14 SNPs), N07_N08 (SNP s19) and N06_N07 (s545), and were highly similar to previously investigated plague victims from other European countries. Thus, beside the assumed continuous reintroduction of *Y*. *pestis* from central Asia in multiple waves during the second pandemic, long-term persistence of *Y*. *pestis* in Europe in a yet unknown reservoir host has also to be considered.

## Introduction

The first detection of the plague bacterium, *Yersinia pestis*, in human skeletons of the Middle Ages in 1998 using PCR [1] led to increasing efforts of scientists in detecting the causative agent of plague in skeletons from both the second plague pandemic of the 14^th^ to 17^th^ century and the first pandemic from the 6^th^ to 8^th^ century, the so called “Plague of Justinian” or “Justinianic Plague”, named after the Byzantinian (eastern Roman) emperor Justinian I. (reign 527 to 565 AD). During both pandemics millions of people were killed. In Europe the second pandemic peaked in the years 1346–53, a time period most commonly known as the “Black Death”, wiping out almost one third of the entire population [2].

Until recently, the etiologies of the Black Death and particularly of the first pandemic were controversially discussed among historians but also among scientists. Some researchers expressed doubts about *Yersinia pestis* having caused these pandemics, but rather a viral disease was discussed [3].

Recent molecular DNA analysis of archaeological skeletal material, however, unequivocally confirmed *Y*. *pestis* to have played a major role in both historical pandemics [4,5,6,7,8]. A scientific milestone was reached in 2011 when the first genome of *Y*. *pestis* from a plague victim of the Black Death was published [6]. However, the study doubted that *Y*. *pestis* also caused the first pandemic. Only as recently as in 2013, *Y*. *pestis* was unambiguously confirmed in plague victims of the first pandemic [7], and the entire genome sequence of one 1500 year-old *Y*. *pestis* strain could be determined [8]. Thus it is now without any doubt that *Y*. *pestis* played a major role during both pandemics. Most recent data on ancient plague provided evidence that plague infection was endemic in the human populations of Eurasia as early as 5, 000 years ago [9]. One of, and presumably the most challenging, still unanswered questions concerning both pandemics is how they could have continued for several hundred years. One theory assumes that the agent of plague has been continuously reintroduced from central Asia to Europe in several waves along the major trade routes, like the Silk Road [10]. Another hypothesis supposes that the agent of plague persisted in Europe for a longer time in a yet unidentified host, e.g. lice [11]. In the latter case identical or very similar genotypes should be present in plague victims from different time periods of both pandemics. In the first case, various different genotypes, reflecting the natural genetic diversity of Asian *Y*. *pestis* strains, should be detectable among different plague victims.

In order to find an answer to this question we analyzed *Y*. *pestis* DNA from plague victims of the second plague pandemic covering a time span of roughly 300 years and originating from different burial sites in Germany, using SNP analysis. Furthermore we compared these data to previously published data from other European countries.

Since SNP analysis allows correct placement of any *Y*. *pestis* strain into the phylogeographic framework of *Y*. *pestis*, it has been used by authors of several recent studies to deeper analyze *Y*. *pestis* from skeletons of the second and the first plague pandemic [4,6,7,8]. However, because historical material is limited and analysis of aDNA requires special facilities and strict precautions in order to avoid contaminations, typing data on historical plague samples is still very limited. Genome sequences of historical plague are available for only one strain of the Black Death period [6] and one strain from the first pandemic [8]. European localities of the second pandemic investigated so far are restricted to the western part of Europe. Our study provides reliable genotyping data from skeletons excavated at Manching-Pichl (Bavaria) and Brandenburg (state of Brandenburg) which represent the most eastern burial sites investigated to date. We demonstrate that a single *Y*. *pestis* genotype persisted in Germany for a period of 300 years during the second plague pandemic and that this genotype also existed in other parts of Europe.

## Material and Methods

### Ethics Statement

In our study we analyzed 300–600 year-old archaeological human remains from two German localities, from Manching-Pichl (MP) and Brandenburg (B).

The designations of the investigated samples are: MP03-I, MP10-I, MP17-I, MP19-II, MP22, MP26-I, MP34-I, MP54-VI, MP56-II, MP59-I, MP73-I, MPS01-I, MPS03-IIX, MPS04-VI, MPS08-XXXI, MPS09-II, MPS09-VI, MPS12-I, MPS15-V, MPS15-VII, MPS4-XX, B1, B2 and B3.

Two of the coauthors, Prof. Dr. Gisela Grupe and Dr. Michaela Harbeck, are representatives of the State Collection for Anthropology and Palaeoanatomy, Munich, Germany. The State Collection is the responsible authority of the Federal State of Bavaria for conservation of and scientific research on archaeological skeletal remains found in Bavaria, for this study for all samples from Manching-Pichl.

The samples from Brandenburg were provided and investigation was authorized by the official authority “Brandenburg Landesamt für Denkmalpflege und Archäologisches Landesmuseum“. They belong to a government agency of the Federal State of Brandenburg, whose mission is the preservation of the cultural and historical heritage of Germany.

The samples from Basel were provided by the research department “Archäologische Bodenforschung Basel-Stadt”. It is affiliated to the Department of Presidential Affairs of the Canton of Basel-Stadt. Its aim is to protect and care for the canton’s archaeological heritage.

According to the authorities’ rules we did not need further consent or permission to research on the ancient human material.

### Plague victims

**Fig 1:**
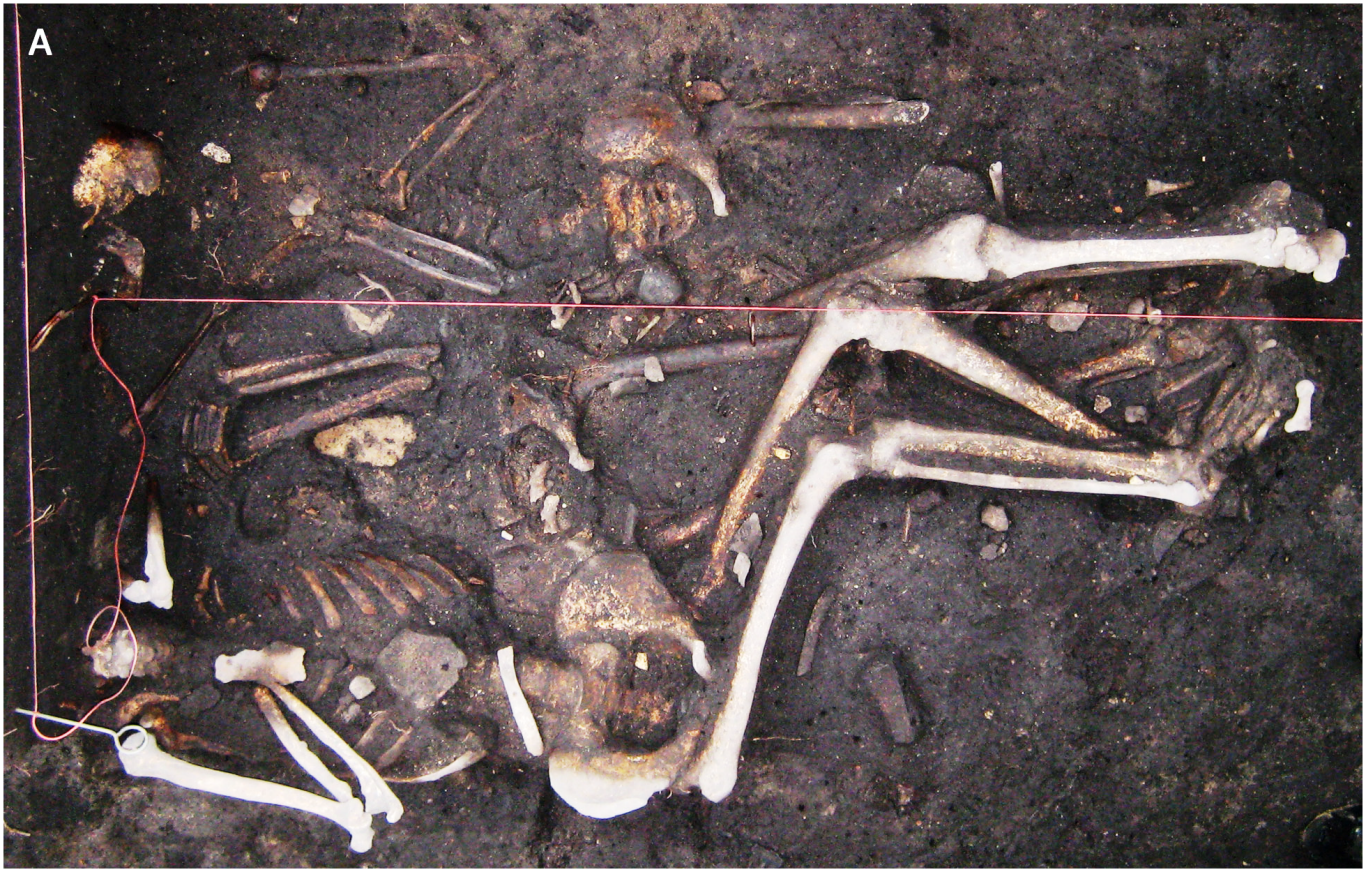
Original photograph of the triple-inhumation regarding the three male soldiers (Brandenburg, Germany), dated to the Thirty Years’ War (1618–1648).

Of 30 previously investigated skeletons [12], 8 individuals were positive for *Y*. *pestis*-specific nucleic acid. Six of the positively tested samples showed ct-values of <35 in the *pla*-specific qPCR and could be used for further SNP analysis. Five of these individuals belonged to a mass grave beneath the sacristy of the St. Leonhard Catholic church in Manching-Pichl, southern Germany [13]. They were assigned to the 14^th^ century by radiocarbon dating (conducted by the Curt-Englhorn-Zentrum Archeometrie gGmbH) [14]. One sample originated from a multiple inhumation of three male soldiers located in Brandenburg, northeastern Germany (Fig 1). This inhumation was dated to the Thirty Years’ War (1618–1648) [15].

### SNP typing of ancient *Y*. *pestis*

**Fig 2:**
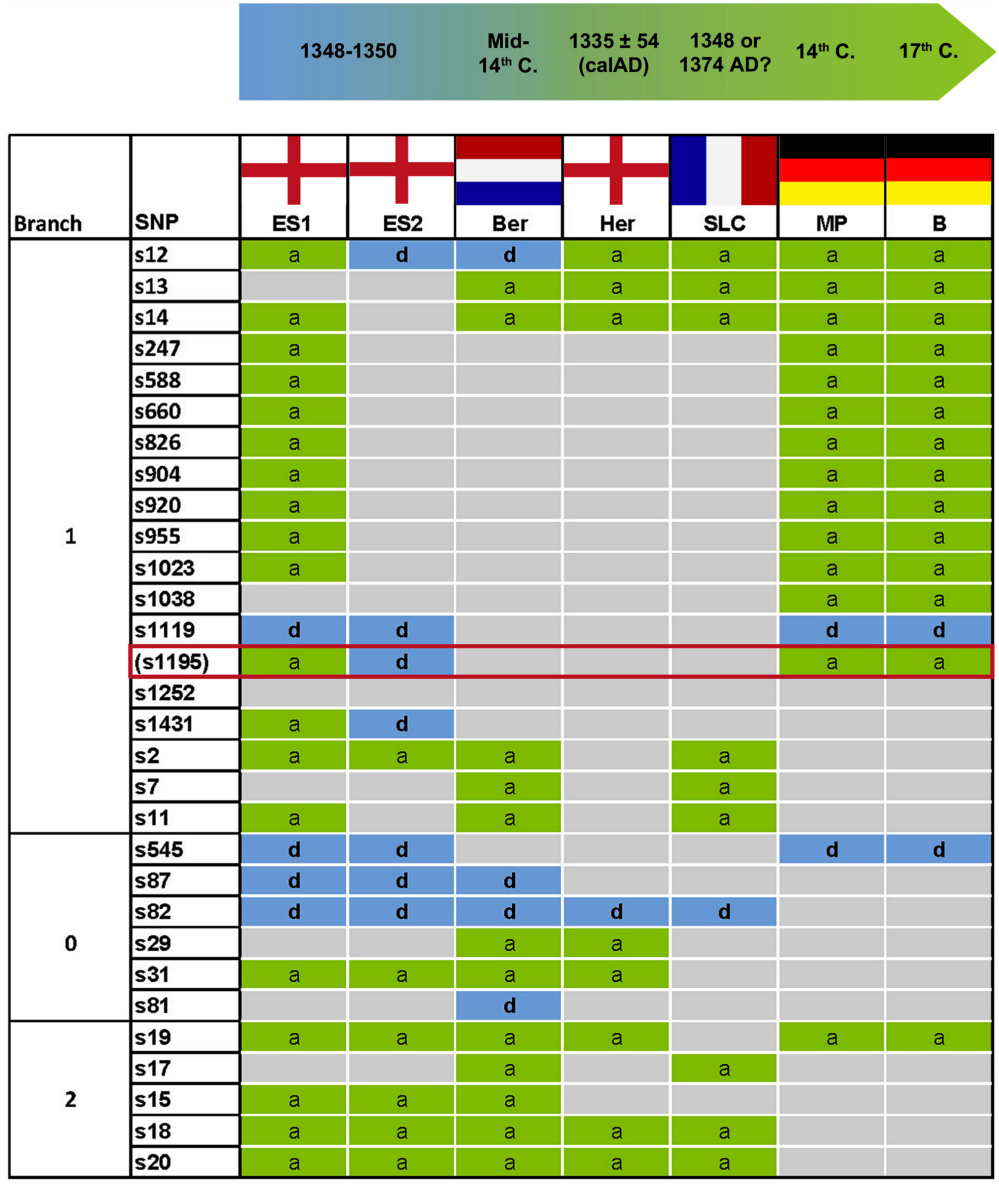
Illustration of *Y*. *pestis* SNP typing results among European victims during three centuries. The flags indicate the sample origin. ES: East Smithfield, London, England; Ber: Bergen op Zoom, the Netherlands; Her: Hereford, England; SLC: Saint-Laurent-de-la-Cabrerisse, France; MP: Manching-Pichl, Southern Germany; B: Brandenburg, Northern Germany. The distance between locations MP and B is 500 km. ES1 and ES2 refer to two different genotypes found at one location. Branches 0, 1 and 2 refer to a previously published phylogenetic tree [16]. SNPs are designated to ancestral (a: green), derived (d: blue) or undetermined status (grey). The ES1-genotype was found in three human individuals, the ES2-genotype in only one; the Dutch Ber-genotype was found in six human individuals, the English Her-genotype in two, the French SLC-genotype in one [4,6]. In the present study, the German MP-genotype was detected in four human individuals, the German B-genotype in one individual. SNP s1195 (red box) is located at position 2,896,636 in the genome of *Y*. *pestis* Type strain CO92 (Genbank AL109969.1) [16]. The SNP is located within the 48bp repetitive and variable region R0664 (2,896,594–2,896,641), and was therefore recently excluded from further phylogenetic interpretation [16].

**Fig 3:**
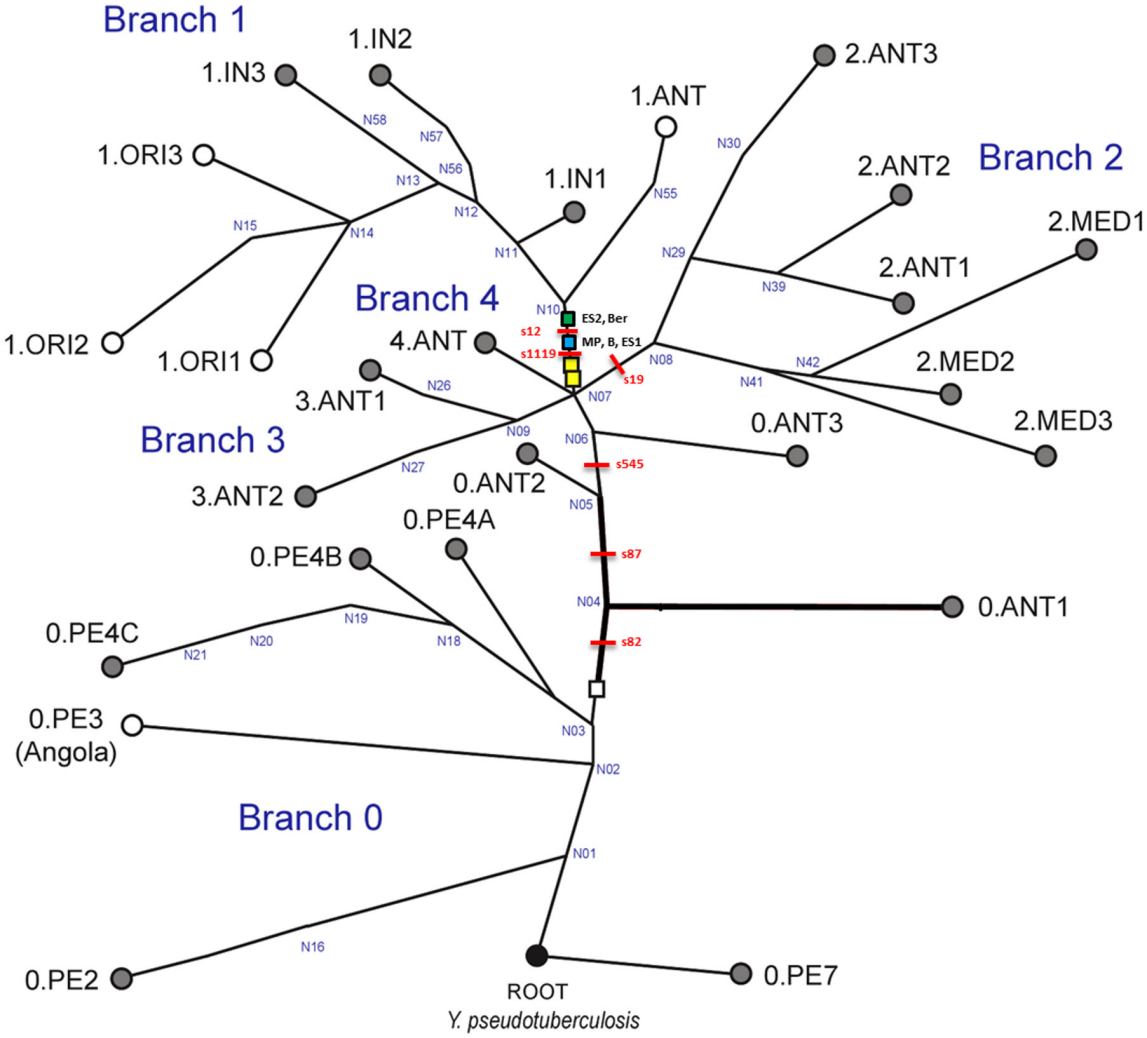
Global phylogeny for *Y*. *pestis*. This global phylogeny for *Y*. *pestis* is based upon fig 1A and S3B in Cui et al. [16]. It includes four major branches (0–4) and is rooted with *Y*. *pseudotuberculosis*, the ancestor of *Y*. *pestis*. Key SNPs s545 and S19 previously identified in studies [16, 17] separate branch 0 and branch 2 from branch 1, respectively. All strains were derived for s545 and ancestral for S19, indicating their location on branch 1. A total of 14 SNPs between nodes N07 and N10 were tested. The derived state of samples ES2 and Ber at position s12 (as previously described in 4) places them closer to node N10. Samples from this study (Mp and B) and ES1 have the derived state of SNP s12, which maps them closer to N07 at the beginning of branch 1.

Genotyping of *Y*. *pestis* from skeletal remains was conducted as recently published, obeying strict anticontamination and decontamination protocols [12]. In addition, SNPs were confirmed by sequencing of the generated PCR products (not shown). No contamination was detected during this study. For typing, 16 SNPs were selected from the previously published most ancestral phylogenetic branch 0 (Nodes N06_N07: SNP s545), branch 1 (Nodes N07_N10: 14 SNPs), and branch 2 (Nodes N07_N08: SNP s19) (Figs 2 and 3) according to Cui et al. [16]. PCR protocols and conditions are given in Table 1 and details on the primer sequences are provided as supplementary material (S1 Table).

**Table 1:** PCR protocols and conditions.

Conventional PCR (final volume 50 μl)						
Reaction mixtures			Cycling conditions			
Substance	Concentration	Manufacturer*	Step	Temperature	Time	Cycles
Multiplex PCR Master Mix	1x	Qiagen, Hilden	Uracile cleaving	25°C	10 min	1x
UDG	0.01 U/μl	Roche, Mannheim	PCR activation	95°C	15 min	1x
BSA	0.4 mg/ml	Ambion/ Life Technologies, Darmstadt	Amplification	94°C	30 sec	50x
Forward and reverse primer	see S1 Table	TibMolbiol, Berlin	Annealing	See S1 Table	30 sec	
2.0 to 4.0 μl DNA	n. d.		Elongation	72°C	60 sec	
			Final elongation	72°C	10 min	
			Cooling	8°C	Until analysis	
**qPCR (final volume 12 or 24 μl)**						
Platinum^®^ Quantitative SuperMix-UDG	1x	Invitrogen/ Life technologies, Darmstadt	Uracile cleaving	50°C	2 min	1x
BSA	0.4 mg/ml	Ambion/ Life Technologies, Darmstadt	PCR activation	95°C	10 min	1x
Forward and reverse primer	0.9 μM	TibMolbiol, Berlin	Amplification	95°C	10 sec	50x
Probes	See S1 Table	TibMolbiol, Berlin	Annealing temperatures	See S1 Table		
MgCl2	3mM (s19), 5mM (s12), 6mM (s545)	Applied Biosystems, Life Technologies, Darmstadt	Cooling	4°C	30 sec	1x
2.0 or 4.0 μl DNA	n. d.					

^§^ for individual annealing temperatures see S1 Table

* for primer sequences see S1 Table

All molecular genotyping assays were adjusted to the ancient material and thoroughly validated as described in [12].

## Results

The complete SNP typing (16 positions) was successful and reproducible for four individuals from Manching-Pichl (MP) and one historic individual from Brandenburg (B) (Fig 2). Despite a geographical distance of 500 km and in particular a time difference of 300 years, an identical *Y*. *pestis* genotype was detected in all five human individuals (Fig 2). Compared with published data [4,6] the SNP pattern was also identical to the genotype of three individuals from the East Smithfield (ES) cemetery in London, UK, dated to 1348–1350 (Figs 2 and 3). However, beside the genotype that persisted for 300 years, three deviant SNPs, s12 and s1431, and s1195 had been described in earlier studies among victims of the second pandemic [4,6], indicating the presence of further genotypes. Nucleotide sequencing of the s1195-specific amplicons during this study revealed a repetitive region, confirming the results of a recent study [16]. As this particular SNP-position changes its derived to an ancestral state, depending on the number of repeats, it has recently been excluded from further phylogenetic interpretation [16].

## Discussion and Conclusions

The identical *Y*. *pestis*-SNP genotype among plague victims from the 14^th^ and the 17^th^ century suggests a possible long-term persistence of one distinct genotype in Germany in a geographical distance of more than 500 km. Furthermore, the SNP data generated from five individuals of the present study are fully consistent with those of three individuals from the cemetery East Smithfield, London (ES 8291, ES 11972, ES 8124) and those of individuals from Herford (Her) und Saint-Laurent-de-la-Cabrerisse (SLC) (Fig 2) [4,6]. Therefore, the *Y*. *pestis* genotype detected in the German victims, positioned between nodes N07 and N10 on major branch 1 of the *Y*. *pestis* phylogenetic tree (Fig 3), suggests a larger geographic distribution of this particular genotype in Europe. Presumably, also the *Y*. *pestis* genotypes from Her and SLC individuals are identical or at least highly similar to the genotype of the present study. Final phylogenetic interpretation, however, will require additional SNP analyses or whole genome sequencing of *Y*. *pestis* from various plague victims. Like the majority of the investigated individuals, the German genotype differed from the strain that was found in the fourth analyzed individual, ES 6330, and from the one detected in individuals from Bergen op Zoom (Ber) (Fig 2) [4,6]. Those are more closely related to strains of branch 1, as indicated by a derived state of s12 (Fig 3). In conclusion, thus far only two different genotypes of *Y*. *pestis* have been detected among plague victims of the second pandemic in Europe of which one, described in the present study, covers a time period of 300 years. This finding is new and raises several questions.

Based on their previous findings, Haensch *et al*. suggested that plague was imported to Europe from Central Asia on at least two occasions by distinct trading routes [4]. Other authors further assumed that the bacterium was continuously reimported into Europe during the second plague pandemic, instead of believing in the establishment of rodent plague reservoirs, in which the agent could have persisted for a longer time [10].

In contrast to these hypotheses, the results of the present study clearly indicate that at least one genotype, which was introduced to Europe at the beginning of the Black Death from Asia, persisted in Europe from the 14^th^ century until the Thirty Years’ War (1618–1648). We therefore suggest a model in which *Y*. *pestis* was introduced to Europe from Asia in several waves combined with a long-time persistence of the pathogen in not yet identified reservoirs.

## Supporting Information

S1 Table Primers and probes used in the present study. (DOCX)
