# Supplementary material for: Genotyping Yersinia pestis in Historical Plague: Evidence for Long-Term Persistence of Y. pestis in Europe from the 14th to the 17th Century
Source: PLoS One. 2016 Jan 13;11(1):e0145194. doi: 10.1371/journal.pone.0145194 (PMC4712009; doi:10.1371/journal.pone.0145194)
Supplement: S1 Table — (DOCX) [file pone.0145194.s001.docx]

**S1 Table. Primers and probes used in the present study**

| **SNP** | **Primer/ probe** | **Sequence 5’-3’** | **Amplicon size (bp)*** | **Conc (µM)** | **AT (°C)**^§^ |
| --- | --- | --- | --- | --- | --- |
| s12** | s12S | CCCgATTggTgATgACgAAgATT | 69 (23) | 0.9 | 58 |
|  | s12R | AgCggTAgCTCCAgAgTggTATC |  | 0.9 |  |
|  | s12 TM wt | FAM-AATCgCCCAgATgT-BBQ |  | 0.2 |  |
|  | s12 TM mut | YAK-AATCACCCAgATgT-BBQ |  | 0.4 |  |
| s13 | s13_fwd_IMB | GTCTGCCAATGGATATTTCTG | 122 (73) | 0.2 | 56 |
|  | s13_L4^*^ | CGAAAATGGGTGTAAAAGCGAAAT |  | 0.2 |  |
| s14 | s14_fwd_IMB | CATTAACAGCAATCCCATTAACCG | 121 (76) | 0.2 | 56 |
|  | s14 _U1^*^ | GGTGAGGTAGTCGTCGTTTGTG |  | 0.2 |  |
| s247 | s247_fwd | CAAATAATAGGCAGTCAGCCC | 123 (85) | 0.2 | 60 |
|  | s247_rev_IMB | GCTCTCGGTGCTTACAGCGA |  | 0.2 |  |
| s920 | s920_fwd_mo^†^ | ATTAGCCAGTGCCGGTAATC | 117 (70) | 0.2 | 56 |
|  | s920_rev_IMB | GTTAATATTTTACTGCCACTGACTGC |  | 0.2 |  |
| s588 | s588_fwd_IMB | GTCGGTGAATAAGCTCACGAAG | 122 (81) | 0.2 | 62 |
|  | s588_rev_mo^†^ | GATACCCACTCGGCCAAAC |  | 0.2 |  |
| s1119 | s1119_fwd_IMB | TTAATTAATGTTTTTTTCATAAGGAGG | 131 (75) | 0.2 | 56 |
|  | s1119_rev_IMB | ATTCATAACTAATAAACATTTGTGCGAG |  | 0.2 |  |
| s955 | s955_fwd_mo^†^ | GCCTTTTCTTTTTCGGTGAC | 122 (71) | 0.2 | 56 |
|  | s955_rev_IMB | GGTATTGTAGGTAATGTCAGCTATGTAC |  | 0.2 |  |
| s904 | s904_fwd_IMB | CGGCCTAATTCGGCGATAC | 125 (84) | 0.2 | 56 |
|  | s904_rev_mo^†^ | TCGGTATCGGTTTCCCATTG |  | 0.2 |  |
| s1038 | s1038_fwd_IMB | GCCTCATGACTTCCAACTGCG | 127 (71) | 0.2 | 62 |
|  | s1038_rev_IMB | CATTTGGAAATAGCGACGATCA |  | 0.2 |  |
| s826 | s826_fwd_IMB | GCACTCAGTGGGGCGCTGAC | 120 (71) | 0.2 | 64 |
|  | s826_rev | CGCTTTGTCGGCTTTTCGTA |  | 0.2 |  |
| s1195 | s1195_fwd_mo^†^ | AGAATGGAGACGATAACGGC | 126-174 (76-118)^‡^ | 0.2 | 56 |
|  | s1195_rev4_IMB | TATTGGATATTGTGATCAATAATGGG |  | 0.2 |  |
| s1023 | s1023_fwd_mo^†^ | TTGACGAACCCCACTTTTCC | 120 (69) | 0.2 | 56 |
|  | s1023_rev_IMB | GGTTCAGATTACGAGAAAATTAGAC |  | 0.2 |  |
| s660 | s660_fwd_mo^†^ | GTTGTGCTGCACTCTTGATG | 116 (66) | 0.2 | 56 |
|  | s660_rev | GTCAGAAATAAAATCATAATTCCCC |  | 0.2 |  |
| s545** | s545 F | ATGCAGACCTGCTTCCTGAAAG | 76 (28) | 0.9 | 62 |
|  | s545 A | CCAGATAGTTAAGAAAGCTGTACGTG |  | 0.9 |  |
|  | s545 TM wt | 6FAM-CAGCGCAGTCTCCCCG--BBQ |  | 0.4 |  |
|  | s545 TM mut | YAK-TCAGCACAGTCTCCCCGACT-BBQ |  | 0.45 |  |
| s19** | s19S | GGATGTGGATCGGGACTTTC | 78 (38) | 0.9 | 58 |
|  | s19A | CTTGTGGCAGATTGGCATCC |  | 0.9 |  |
|  | s19TM mut | YAK--TGCTACGTGCTCACCATCTGG--BBQ |  | 0.4 |  |
|  | s19TM wt | 6FAM-TGCTGCGTGCTCACCATC--BBQ |  | 0.3 |  |

^*^reference [4]; ^§^AT: annealing temperature; ** endpoint genotyping using real-time technique

^†^ reference [12]; ^‡^ SNP s1195: amplicon size varies depending on the number of repetitive elements
